# Optic nerve head optical coherence tomography angiography findings in patients with thyroid eye disease: a case–control study

**DOI:** 10.1186/s13044-022-00134-4

**Published:** 2022-09-21

**Authors:** Aliakbar Sabermoghaddam, Nasser Shoeibi, Hamid Jafarzadeh, Elham Bakhtiari, Zeinab Salahi, Talieh Saeidi Rezvani, Hamid Reza Heidarzadeh, Mojtaba Abrishami

**Affiliations:** 1 Eye Research Center, Mashhad University of Medical Sciences, Mashhad, Iran; 2 Department of Education and Psychology, Ferdowsi University of Mashhad, Mashhad, Iran

**Keywords:** Thyroid eye disease, Optical coherence tomography angiography, Optic nerve head, Radial peripapillary capillary network

## Abstract

**Background:**

To evaluate changes in the vessel density (VD) of the optic nerve head (ONH) microvasculature in thyroid eye disease (TED) using optical coherence tomography angiography (OCTA). This study aimed to applicate the OCTA as a noninvasive modality in screening TED patients to assess sub-clinical changes.

**Methods:**

In a cross-sectional study, the control group patients were healthy individuals with no ocular abnormalities and were euthyroid. All patients with TED had clinical features of the disease. We divided them into two groups using the clinical activity score (CAS). Patients with CAS scores 0–2 were categorized as group A, and scores three or more as group B. All vessels (AV) and small vessels (SV) VD inside disc and radial peripapillary capillary network were measured using the ONH-OCTA.

**Results:**

We evaluated 29 patients with TED and 28 healthy controls. The mean whole image AV VD (mean ± SD: 56.33 ± 2.56, *p*-value = 0.17) and the mean whole image SV VD (mean ± SD: 49.94 ± 2.56, *p*-value = 0.16) in the TED group had no statically significant difference compared with the control group (AV mean ± SD: 57.20 ± 20.22, SV mean ± SD: 50.84 ± 2.23). We found a non-significant decrease in AV and SV radial peripapillary capillary VD in the TED group. There was a significant decrease in the mean whole image AV VD (mean ± SD: 54.83 ± 3.07, *p*-value = 0.005) and the mean whole image SV VD (mean ± SD: 48.60 ± 3.18, *p*-value = 0.013) in CAS group B compared to group A (AV mean ± SD: 57.45 ± 1.33, SV mean ± SD: 50.95 ± 1.37).

**Conclusion:**

Our study showed non-significant ONH vascular alterations in patients with TED, including reduced VD of ONH in the radial peripapillary capillary. Patients with higher CAS scores had a more noticeable decrease in ONH microvasculature.

## Background

Thyroid eye disease (TED) is one of the main extra-thyroidal manifestations of Graves' disease [1]. Although it often develops in patients with hyperthyroidism, it can also occur in association with euthyroidism or hypothyroidism [2]. Subclinical eye involvement in Graves' disease is prevalent, and half of Graves' disease patients present with the spectrum of TED. In these patients, orbital fibroblasts and adipocytes are targeted by auto-immune reactions. It leads to edema and inflammation of extraocular muscles and increases physical pressure on the orbital connective tissue and fat [3]. Changes in TED are usually documented in the orbital and periocular tissues, and the activity of the disease is usually characterized by clinical findings [3]. Optic nerve compression is the result of increased pressure in the orbital cavity. It leads to ischemia and nerve damage in the affected eye [4]. However, in some cases, optic nerve involvement occurs without prominent orbital involvement [4].

The retinal microvascular network was previously evaluated using fluorescein angiography. It is an invasive and time-consuming procedure with two-dimensional images [4]. Optical coherence tomography angiography (OCTA) is a non-invasive technique that gives us high-resolution three-dimensional maps of the retinal and choroidal microvasculature. It also makes it possible to quantify the superficial and deep retinal capillary plexus in the fovea and radial peripapillary capillary network in the peripapillary area [5]. In small case series of patients with TED, significant optic nerve head (ONH) changes have been reported using optical coherence tomography [6–8]. In this study, we aimed to evaluate the density of the ONH microvasculature in TED using OCTA imaging. We may use OCTA to screen patients with TED for sub-clinical compressive optic neuropathy.

## Methods

### Study population

In this cross-sectional case–control study, hyperthyroid patients were enrolled in the study. The diagnosis of hyperthyroidism was confirmed by laboratory tests and endocrinologists. All patients in the TED group had clinical features of TED and documented laboratory test confirmation of hyperthyroidism. The control group patients were healthy individuals with no ocular abnormalities and were euthyroid according to clinical examination and laboratory test results. Cases were selected consecutively in a convenient sampling manner. Detailed ocular and systemic histories were obtained. Exclusion criteria included a history of diabetes mellitus, current pregnancy and breastfeeding, migraine, auto-immune disease, and any intraocular surgeries. Additional exclusion criteria considered as more than five diopters absolute spherical and greater than two diopters cylindrical refractive errors, best-corrected visual acuity less than 20/20, glaucoma, smoking, clinically apparent retinal disease, and other ocular diseases. Patients with a history of dysthyroid optic neuropathy were excluded. Moreover, signs of dysthyroid optic neuropathy like a positive relative afferent pupillary defect, decreased visual acuity, and decreased color vision were checked, and patients with any positive sign for optic neuropathy were excluded from study.

Retinal evaluation performed by a vitreoretinal sub-specialist. Palpebral fissure measurement, exophthalmometry, TED clinical activity score (CAS), and corneal condition evaluated by an oculoplastic sub-specialist.

### Assessment of severity and activity of TED

The severity of TED was scored with a modified NOSPECS classification, and the TED activity was evaluated with a 7-point scale CAS index based on Mourits et al. presented criteria [9]. Each item has one point. CAS is the sum of individual scores, and ranging from 0 (no activity) to 7 (maximal activity). Seven parameters are spontaneous retrobulbar pain, painful eye movements, eyelid erythema, conjunctiva injection, chemosis, caruncle swelling, and edema or fullness of the eyelid. Patients with CAS 0–2 were categorized as group A and scored three or more as group B.

### Imaging procedures

Imaging was performed in the imaging clinic of Khatam al Anbia Eye Hospital from December 2018 to September 2019. The spectral-domain instrument used for optical coherence tomography and OCTA images (AngioVue) is based on the Optovue RTVue XR Avanti technology to obtain optical coherence tomography images with a wavelength of 840 nm and an A-scan rate of 70,000 scans per second. The radial peripapillary capillary network evaluations were acquired using the default automated segmentation with the preset settings. Using optical coherence tomography 3D volume set at 4.5 × 4.5 mm, the AngioDisc 4.5 × 4.5 mm HD scan (400 lines × 400 A-scans) protocols with AngioVue 3D Projection Artifact Removal were applied.

All images centered on the optic disc and scan quality indexes were 7/10 or better. Low-quality images were discarded and reacquired. All images were carefully reviewed for sufficient quality and resolution. Images with significant motion artifacts that interfere with vessel density (VD) analysis were excluded. For all participants, the analysis used eye data with better image quality.

The radial peripapillary capillary network was evaluated using a slab between the outer edge of the retinal nerve fiber layer and the internal limiting membrane. All images were checked for segmentation errors and were adjusted manually before testing the VD. The machine software evaluated all vessels (AV) and small vessels (SV) VD separately in the RPC network. The analyses for the radial peripapillary capillary SV VD were reported as the whole image, inside disc area, whole peripapillary, superior and inferior hemifields, and eight segments. For the evaluation of AV VD, we divided the whole image into nine (three by three) grid-based sections, and it was reported separately in all sections. Moreover, the AV VD was reported for the whole image, inside the disc area, whole peripapillary, and peripapillary superior and inferior hemifields.

### Statistical analysis

The variables' normal distribution was examined using the Shapiro–Wilk test and variances normality plots, and Levene's test determined homogeneity. The independent-samples t-test, paired t-tests, or Mann–Whitney U test was used for comparisons based on data distribution and type. The statistical significance level was set at 0.05. *P*-values were corrected for multiple comparisons. The SPSS program version 16 for Windows was used for all statistical analyses (IBM SPSS Statistics, IBM Corporation, Chicago, IL, USA).

### Ethical considerations

The study protocol adhered to the tenets of the Declaration of Helsinki. All participants provided written informed consent, and the Regional Committee on Medical Ethics approved the ethical aspects of the study at Mashhad University of Medical Sciences, Mashhad, Iran (IR.MUMS.MEDICAL.REC.1397.629).

## Results

**Fig. 1:**
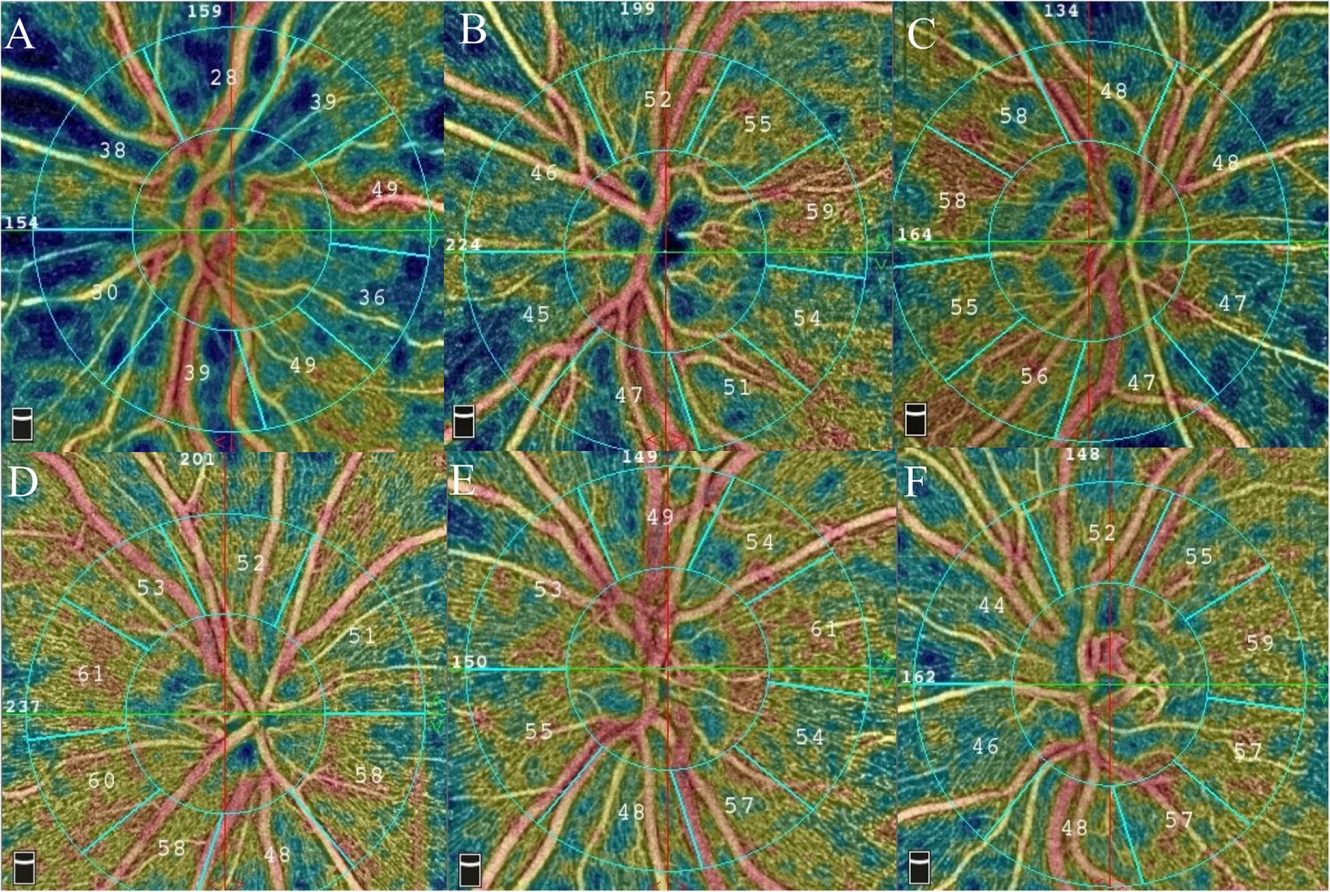
En-face optical coherence tomography angiograms (OCTA) segmented at the level of the radial peripapillary capillary network from three patients with thyroid eye disease (TED) (**A**-**C**) versus three age-matched normal controls (**D**-**F**). Note the remarkable flow deficits present in the en-face angiograms from the TED cases

We enrolled 29 patients with TED (19 females, 65.5%) (29 eyes) and 28 normal healthy controls (18 females, 64.2%) (28 eyes) in the study. The mean ages of the TED and control groups were 42.5 ± 10.7 and 39.7 ± 6.3 years, respectively. The difference in mean ages (*p*-value: 0.146) and the participant's gender (*p*-value: 0.462) between the two groups were not statistically significant. The TED and control groups mean scan quality were 8.11 ± 0.68 and 8.42 ± 0.66, respectively (*p*-value: 0.178) (Fig. 1). Among patients with TED, eighteen were grouped in CAS group A and eleven in group B.

**Fig. 2:**
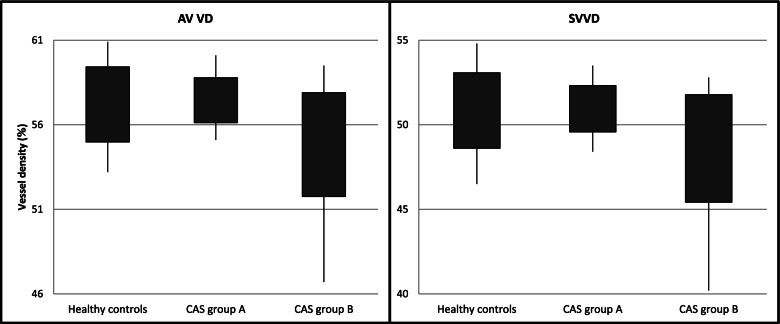
Comparison between different groups all vessel and small vessel vessel density. (AV: All vessel, SV: Small vessel, VD: Vessel density)

The TED group mean 4.5 × 4.5 mm whole image SV VD (49.94 ± 2.56) had no statistically significant difference compared to the control group (50.84 ± 2.23) (*p*-value: 0.16) (Table 1). Also, the TED group mean 4.5 × 4.5 mm whole image AV VD (56.33 ± 2.56) had no statistically significant difference compared to the control group (57.20 ± 20.22) (*p*-value: 0.17); however, there was a tendency for lower values in the TED group (Table 2). In addition, the TED group had non-significant lower means for VD in AV and SV VD in all other parameters compared to the control group (Fig. 2).

**Table 1:** Comparison of the mean small vessels (SV) vessel density (VD) between patients with thyroid eye disease (TED) and normal controls eyes. (SV: small vessels; VD: vessel density; SD: Standard Deviation)

	Healthy controls (28 eyes) Mean ± SD (Range)	TED patients (29 eyes) Mean ± SD (Range)	*P*-value
Whole image SV VD	50.84 ± 2.23 (46.5,54.8)	49.94 ± 2.56 (40.20,53.70)	0.16
Inside disc SV VD	49.39 ± 4.76 (40.40,57.70)	48.44 ± 5.18 (37.50,60.00)	0.47
Whole peripapillary SV VD	53.75 ± 2.65 (48.00,59.30)	52.80 ± 3.38 (38.20,55.90)	0.24
Peripapillary superior hemifield SV VD	54.36 ± 2.59 (48.50,60.00)	53.13 ± 3.38 (38.60,56.50)	0.12
Peripapillary inferior hemifield SV VD	53.06 ± 3.09 (47.00,58.60)	52.42 ± 3.70 (37.70,56.20)	0.48
Peripapillary nasal superior SV VD	51.38 ± 2.88 (43.80,58.00)	50.25 ± 4.68 (38.30, 60.00)	0.27
Peripapillary nasal inferior SV VD	48.98 ± 5.22 (33.00,56.30)	48.15 ± 4.45 (35.90,58.00)	0.51
Peripapillary inferior nasal SV VD	51.95 ± 4.37 (41.40,61.20)	51.41 ± 5.12 (35.90,59.90)	0.66
Peripapillary inferior temporal SV VD	59.03 ± 3.10 (53.10,64.60)	58.26 ± 3.97 (49.80,64.80)	0.41
Peripapillary temporal inferior SV VD	54.94 ± 4.77 (45.5,64.00)	53.40 ± 5.29 (40.60,62.00)	0.24
Peripapillary temporal superior SV VD	57.24 ± 3.48 (51.00,63.40)	55.70 ± 4.19 (46.80,63.40)	0.13
Peripapillary superior temporal SV VD	57.78 ± 2.50 (52.30,62.90)	55.94 ± 4.77 (43.00,62.60)	0.07
Peripapillary superior nasal SV VD	51.55 ± 4.17 (44.20,64.40)	50.55 ± 5.07 (34.90,61.50)	0.41

**Table 2:** Comparison of all vessels (AV), including both small and large vessels, vessel density (VD) of patients with thyroid eye disease (TED) versus normal controls eyes. (AV: all vessels; VD: vessel density; SD: Standard Deviation)

	Healthy controls (28 eyes) Mean ± SD (Range)	TED patients (29 eyes) Mean ± SD (Range)	*P*-value
Whole image AV VD	57.20 ± 2.22 (53.20,60.90)	56.33 ± 2.56 (46.70,61.10)	0.17
Inside disc AV VD	59.28 ± 3.86 (50.10,64.60)	57.97 ± 4.38 (45.50,67.80)	0.23
Whole peripapillary AV VD	59.88 ± 2.47 (55.10,64.20)	59.03 ± 3.14 (45.70,62.00)	0.26
Peripapillary superior hemifield AV VD	60.52 ± 2.41 (55.40,65.00)	59.68 ± 3.08 (46.70,62.80)	0.25
Peripapillary inferior hemifield AV VD	59.18 ± 2.83 (52.50,63.40)	58.32 ± 3.37 (44.70,62.30)	0.29
Grid based superotemporal AV VD	59.86 ± 2.22 (56.20,64.70)	57.94 ± 4.44 (44.00,64.60)	**0.04**
Grid based temporal AV VD	57.74 ± 3.19 (51.70,63.20)	56.62 ± 3.63 (46.40,62.60)	0.21
Grid based inferotemporal AV VD	59.91 ± 2.64 (54.90,64.00)	58.46 ± 4.34 (45.60,66.30)	0.13
Grid based superior AV VD	58.60 ± 3.38 (51.40,64.20)	57.03 ± 5.30 (43.00,63.00)	0.18
Grid based central AV VD	60.04 ± 4.07 (49.90,65.90)	59.62 ± 3.94 (50.30,67.40)	0.69
Grid based inferior AV VD	62.55 ± 3.04 (54.50,67.40)	61.73 ± 3.02 (54.30,66.10)	0.30
Grid based superonasal AV VD	52.75 ± 3.96 (45.30,60.00)	50.95 ± 5.14 (38.90,61.60)	0.14
Grid based nasal AV VD	54.49 ± 4.56 (42.80,64.10)	53.11 ± 4.35 (42.40,59.80)	0.24
Grid based inferonasal AV VD	50.49 ± 4.50 (36.10,57.90)	49.23 ± 4.64 (36.80,60.10)	0.29

There was a significant difference in comparison of the mean 4.5 × 4.5 mm whole image SV VD between the CAS group A (50.95 ± 1.37) and group B patients (48.60 ± 3.18) (*p*-value: 0.013) (Table 3). Also, the mean 4.5 × 4.5 mm whole image AV VD in the group A (57.45 ± 1.33) was significantly higher than in the group B (54.83 ± 3.07) (*p*-value: 0.005) (Table 4).

**Table 3:** Comparison of SV VD of CAS group A versus CAS group B patients. Patients with CAS 0–2 were categorized as group A and scored three or more as group B. (SV: small vessels; VD: vessel density; SD: Standard Deviation; TED: thyroid eye disease; CAS: Clinical Activity Score)

	CAS group A (n:18) Mean ± SD (Range)	CAS group B (n:11) Mean ± SD (Range)	*P*-value
Whole image SV VD	50.95 ± 1.37 (48.40–53.50)	48.60 ± 3.18 (40.20–52.80)	0.013
Inside disc SV VD	49.83 ± 3.88 (43.80–55.80)	46.59 ± 6.24 (37.50–60)	0.103
Whole peripapillary SV VD	53.88 ± 1.38 (51.40–55.90)	51.35 ± 4.63 (38.20–55)	0.092
Peripapillary superior hemifield SV VD	54.12 ± 1.61 (51.40–56.50)	51.80 ± 4.59 (38.60–55.70)	0.072
Peripapillary inferior hemifield SV VD	53.58 ± 2.04 (49.60–56.20)	50.87 ± 4.84 (37.70–55)	0.045
Peripapillary nasal superior SV VD	50.55 ± 3.26 (41.20–54.20)	49.81 ± 6.31 (38.30–60)	0.682
Peripapillary nasal inferior SV VD	49.15 ± 3.12 (43.50–54.40)	46.74 ± 5.71 (35.90–58.00)	0.155
Peripapillary inferior nasal SV VD	52.05 ± 3.73 (46.50–58.30)	50.51 ± 6.71 (35.90–59.90)	0.437
Peripapillary inferior temporal SV VD	58.97 ± 3.65 (49.80–62.30)	57.67 ± 4.53 (51.70–64.80)	0.647
Peripapillary temporal inferior SV VD	54.88 ± 3.80 (48.70–61.10)	51.30 ± 6.48 (40.60–62.00)	0.073
Peripapillary temporal superior SV VD	56.29 ± 3.14 (49.10–60.50)	54.85 ± 5.38 (46.80–63.40)	0.373
Peripapillary superior temporal SV VD	57.17 ± 2.94 (51.30–62.60)	54.20 ± 6.30 (43.00–61.50)	0.152
Peripapillary superior nasal SV VD	51.28 ± 3.33 (42.10–56.20)	49.53 ± 6.89 (34.90–61.50)	0.371

**Table 4:** Comparison of AV VD of CAS group A versus CAS group B patients. Patients with CAS 0–2 were categorized as group A and scored three or more as group B. (AV: all vessels; VD: vessel density; SD: Standard Deviation; TED: thyroid eye disease; CAS: Clinical Activity Score)

	CAS group A (n:18) Mean ± SD (Range)	CAS group B (n:11) Mean ± SD (Range)	*P* Value
Whole image AV VD	57.45 ± 1.33 (55.10–60.10)	54.83 ± 3.07 (46.70–59.50)	0.005
Inside disc AV VD	59.20 ± 2.81 (55.20–64.40)	56.33 ± 5.59 (45.50–67.80)	0.087
Whole peripapillary AV VD	60.31 ± 1.25 (57.60–62)	57.33 ± 4.08 (45.70–61.10)	0.010
Peripapillary superior hemifield AV VD	60.86 ± 1.30 (57.90–62.80)	58.10 ± 4.03 (46.70–61.60)	0.016
Peripapillary inferior hemifield AV VD	59.70 ± 1.66 (56.80–62.30)	56.49 ± 4.21 (44.70–60.60)	0.010
Grid based superotemporal AV VD	58.57 ± 3.27 (49.80–63.30)	57.05 ± 5.77 (44.00–64.60)	0.375
Grid based temporal AV VD	56.90 ± 3.17 (52.60–62.60)	56.21 ± 4.31 (46.40–61.70)	0.623
Grid based inferotemporal AV VD	58.91 ± 3.46 (50.70–62.70)	57.84 ± 5.46 (45.60–66.30)	0.523
Grid based superior AV VD	57.89 ± 5.82 (43–63)	55.80 ± 4.41 (47.30–61.70)	0.306
Grid based central AV VD	60.40 ± 2.51 (56.10–65.50)	58.52 ± 5.29 (50.30–67.40)	0.272
Grid based inferior AV VD	61.81 ± 2.37 (57.80–65.30)	61.61 ± 3.87 (54.30–66.10)	0.875
Grid based superonasal AV VD	52.01 ± 5.03 (40.60–61.60)	49.45 ± 5.13 (38.90–57.60)	0.193
Grid based nasal AV VD	55.06 ± 3.06 (48.90–59.80)	50.35 ± 4.50 (42.40–57.10)	0.002
Grid based inferonasal AV VD	50.97 ± 4.32 (43.20–60.10)	46.77 ± 4.06 (38.60–52.10)	0.014

## Discussion

Twenty-nine TED patients were compared with twenty-eight healthy controls to evaluate OCTA VD changes. According to the results, patients with TED were not different from normal subjects in VD, either in SV or AV VD. This study evaluated the OCTA as a noninvasive modality in screening patients with TED for sub-clinical compressive optic neuropathy.

The concern of ONH changes in patients with TED has been considered recently. Increased intra-orbital pressure and extraocular muscle volume could lead to compressive optic neuropathy [10]. It has been reported that optic disc function may be affected before the clinical complaint of decreased vision, and therefore the diagnosis of optic nerve damage initiation could be beneficial in preventing significant visual loss. Therefore, follow-up of patients with hyperthyroidism, especially in visual function, is recommended in the literature [11, 12]. Using diffusion-tensor imaging in computed tomography, the ONH was affected by TED before the development of dysthyroid optic neuropathy compared to controls and between active and inactive stages of TED [4].

Few studies evaluated the ONH parameters in patients with TED compared to healthy subjects. A case–control study by Mihailovic et al. on 29 patients with TED and 29 healthy controls using OCTA, reported that ONH parameters were significantly lower in TED group compared to controls [7]. In another study, patients with TED had thinner, inferior retinal nerve fiber layer thickness. Also, the disc area and cup/disc ratio were higher in patients with TED compared to the control group [6]. The results of our study did not fully support Mihailovic and Sayin's conclusions since we report that the radial peripapillary capillary or disc parameters were not significantly different in TED compared to controls, although the trend was toward decreasing VD. It may be due to the limitation of our study to patients with 20/20 vision, and in Mihailovic et al. study, visual acuity limitation was not considered. Moreover, in that study, only inactive TED cases were enrolled, and they concluded that CAS is not associated with VD.

In our study we have included different severity groups of patients with TED (CAS groups A and B) and we found a decrement in VD with the increase in activity of the TED. The findings of our study showed that in contrast to the anticipation of increasing VD due to engorgement resulting from orbital inflammation, there is decreased vascularity. This finding may present another justification for nerve damage during TED.

It has been proposed that nearly 90% of dysthyroid optic neuropathy cases are associated with nerve compression; the remainder is proposed to be associated with stretching of the optic nerve without compression [13]. A comparative case series reported that although the optic nerve stretching is essential in dysthyroid optic neuropathy, ONH involvement is most probably due to ONH microvascular ischemia secondary to orbital apex changes [13, 14]. Moreover, the main risk factors for dysthyroid optic neuropathy include advancing age, smoking, and diabetes mellitus, which are associated with microvascular changes in OCTA [5, 13]. In a case report, a patient with worsening TED complained of complete vision loss in the left eye on up-gaze, considered gaze-evoked amaurosis [15]. The mechanism of vision loss in this patient was presumed optic nerve ischemia due to elevation in intraocular pressure in a congested optic disc. An increase in choroidal thickness in patients with TED indicates vascular tissue involvement in Graves’ disease. These findings support the vasculogenic pathogenesis of dysthyroid optic neuropathy in TED.

In a study by Ceylanoglu et al. they demonstrate that smoker patients with inactive TED had significantly lower ONH VD compared to healthy controls [16]. So, we aimed to exclude smoker subjects in our study to evaluate the sole effects of TED on ONH VD more specifically.

Yu et al. evaluated the difference in retinal nerve fiber layer thickness, choroidal thickness, and VD between patients with different severities of TED and healthy controls. They divided patients into three groups (active TED, inactive TED, and control groups). They found that VD had no significant relation with different clinical variables [17]. Jian et al. also found that the ONH VD was lower in patients with TED who had optic neuropathy [18]. In another study by Del Noce et al., there was a significant decrease in the peripapillary choriocapillaris and deep capillary plexus VD in patients with TED compared to healthy age and gender-matched controls. The study demonstrates a significant relationship between higher CAS scores and lower peripapillary choriocapillaris VD [19]. These studies showed significant alteration in ONH VD in patients with higher CAS scores like ours.

On the other hand, Pinhas et al., in a study on eight patients with TED, compared the non-capillary and capillary ONH VD with 133 normal eyes. They found that the non-capillary VD decreased significantly in the TED group, but the capillary VD did not show a significant difference. However, their study population was tiny [20].

Decreased vessel density in patients with higher CAS could support the theory that inflammation leads to atrophic changes; hence, perfusion of atrophic tissues is lower because of decreased consumption of oxygen and other nutrients [7]. In patients with active TED, Wu et al. found that the activity status and serum antibodies associated with TED were the relevant factors for reduced capillary density of the retina [8]. These findings correlate the activity of TED with a reduction in retinal or ONH VD, indicating retina lower demand in patients with active disease or retinal ischemia.

We enrolled a relatively small sample size of nearly thirty patients in our study, and this study limitation could improve by a larger-scale OCTA analysis performed in the active phase of the TED. Repeating imaging at fixed intervals in a longitudinal study could provide valuable information about the effects of TED on the ONH vascular system in the short and long term.

## Conclusions

In conclusion, our study showed non-significant ONH vascular alterations in TED compared to healthy controls, including reduced VD of ONH in the radial peripapillary capillary. However, patients with higher CAS scores had a significant decrease in ONH microvasculature. TED's potential vascular involvement of the ONH warrants further study on a larger scale.

## Data Availability

The datasets generated and analyzed during the current study are available from the corresponding author on reasonable request.

## Abbreviations

VD: Vessel density
ONH: Optic nerve head
TED: Thyroid eye disease
OCTA: Optical coherence tomography angiography
AV: All vessels
SV: Small vessels
CAS: Clinical activity score
